# Enhanced citric acid production via aconitase co-inhibition strategy using L-cysteine HCl-resistant *Aspergillus niger* NA-CYS3

**DOI:** 10.1007/s13205-026-04929-2

**Published:** 2026-06-27

**Authors:** Nudrat Jabeen, Mahmood Ul Hassan, Sikander Ali, Aneeba Rashid, Luiza Cintra Campos

**Affiliations:** 1 Dr Ikram-ul-Haq Institute of Industrial Biotechnology (IIB), GC University Lahore, Lahore, 54000 Pakistan; 2 Department of Botany, Dr. Nazir Ahmad Institute of Biological Sciences, GC University Lahore, Lahore, 54000 Pakistan; 3 Centre for Urban Sustainability and Resilience, Department of Civil, Environmental and Geomatic Engineering, University College London, London, WC1E 6BT UK

**Keywords:** Citric acid production, *Aspergillus niger*, NA-CYS3, Continuous fermentation, Aconitase co-inhibition, Nitrous acid mutagenesis

## Abstract

The present study presents a new approach to boost citric acid production by combining genetic improvement and enzyme inhibition in the fungus *Aspergillus niger*. A mutant strain, named NA-CYS3, was developed by chemically treating the wild-type strain to make it resistant to L-cysteine HCl, a chemical that normally limits growth. This mutant showed better tolerance and higher productivity. Optimal fermentation conditions including medium volume (50 mL), acidity (pH: 4.5), inoculum size (10%), incubation time (144 h), and temperature (30 °C) were optimized to maximize citric acid yield. Under these conditions, NA-CYS3 produced 26.35 g/L, a 2.7-fold increase in substrate use and higher yield compared to the wild-type strain. To further increase production, the activity of aconitase, an enzyme involved in the citric acid cycle, was partially blocked by adding two inhibitors, potassium ferrocyanide (*K₄Fe(CN)₆*; 0.004%) and methanol (1 mL), shortly after fermentation started. This “aconitase co-inhibition strategy” slowed down certain metabolic steps, leading NA-CYS3 to produce nearly 49 g/L of citric acid, significantly more than the wild-type’s 35.65 g/L. Kinetic parameters (Yp/s, Qp, qp) further confirmed its superior production capacity. These findings demonstrate that combining strain mutation with targeted enzyme inhibition can greatly enhance citric acid biosynthesis. The NA-CYS3 strain shows promise for efficient and large-scale industrial production of citric acid.

**Supplementary Information:**

The online version contains supplementary material available at 10.1007/s13205-026-04929-2.

## Introduction

Citric acid (2-hydroxypropane-1,2,3-tricarboxylic acid), a weak organic acid, is ubiquitous in nature. Naturally, it occurs in citrus fruits particularly in members of family Rutaceae and some other vegetables (Belen et al. 2010). Biochemically, it is a key intermediate in the Tricarboxylic acid (TCA) cycle, where carbohydrates are oxidized to CO_2_ via the acetyl coA pathway in aerobic organisms. Due to its widespread applications in food, pharmaceutical, and chemical industries, citric acid has become one of the most extensively produced organic acids through microbial fermentation (Show et al. 2021; Behera 2020).

Among various microbial producers, *Aspergillus niger* remains the organism of choice for industrial-scale citric acid production. Although several other fungi and yeasts, including *A. flavus*, *A. saitoi*, *A. phoenicus*, *A. nidulans*, *A. fonsecaeus*, *A. luchensis*, *M. piriformis*, *Absidia* spp., *Botrytis* spp., *Talaromyces* spp., *T. viride*,* Candida* spp., *Torula* spp., *Pichia* spp., *Yarrowia lipolytica* and *Debaromyces* spp., have been explored for its production (Zheng et al. 2022; Adeoye et al. 2015; Papagianni 2007; Vandenberghe et al. 1999), *A.* *niger* is favoured due to its saprotrophic nature, fast growth rate, and high citric acid yield (Nadeem et al. 2020; Madhusudan et al. 2010). Recent advancements have focused on improving *A. niger* strains using classical mutagenesis, CRISPR-based editing, and adaptive evolution (Cairns et al. 2021; Khandagale et al. 2021; Yousuf et al. 2022). Notably, chemical mutagenesis using agents such as ethyl methanesulfonate (EMS), nitrous acid (HNO₂), and N-methyl-N′-nitro-N-nitrosoguanidine (MNNG) continues to serve as a low-cost and effective strategy to enhance metabolite production (Khandagale et al. 2021; Xu et al. 2021; Mazutti et al. 2020). *A. niger* remains the organism of choice for industrial-scale citric acid production (Behera 2020; Zhang et al. 2019; Zheng et al. 2022).

Citric acid biosynthesis in *A. niger* is primarily driven by citrate synthase, which catalyzes the irreversible condensation of oxaloacetate with acetyl-CoA. However, aconitase (EC 4.2.1.3), which converts citrate to isocitrate via cis-aconitate, represents a key branch point enzyme that may reduce citrate accumulation if not regulated. Therefore, downregulating aconitase activity while maintaining or enhancing citrate synthase levels is a logical target for metabolic intervention (Yu et al. 2021). Although strategies like overexpression of biosynthetic genes and metabolic flux redirection have improved productivity (Zheng et al. 2022), specific biochemical inhibition of aconitase remains underexplored.

This study introduces a novel biochemical dual approach that combines strain improvement via chemical mutagenesis and aconitase co-inhibition using potassium ferrocyanide and methanol. This dual strategy has not been previously reported for citric acid fermentation. Methanol induces oxidative stress, potentially altering central metabolic fluxes toward citrate accumulation, while potassium ferrocyanide, a known inhibitor of iron–sulfur cluster enzymes, selectively targets aconitase activity. Additionally, the research explores strain improvement via chemical mutagenesis to isolate a L-cysteine HCl-resistant auxotrophic mutant, designated *A. niger* NA-CYS3 using HNO₂ mutagenesis. This combined metabolic and physiological strategy offers an efficient and scalable model for improving organic acid production.

The objectives of this work include: (i) developing a chemically mutated high-yielding strain through nitrous acid mutagenesis; (ii) optimizing fermentation parameters for citric acid production; (iii) applying aconitase co-inhibition to enhance productivity; and (iv) evaluating fermentation kinetics including substrate utilization, biomass accumulation, and product yield. The findings are expected to contribute to cost-effective and sustainable strategy for enhanced industrial citric acid production.

## Materials and methods

### Chemicals

The chemicals viz. dinitro salicylic acid (DNS), phenol, coomassie G-250, phosphoric acid and potassium ferrocyanide of laboratory grade purity procured from E-Merck (Germany) and Sigma Chemicals Inc. (USA). All the other chemicals have been procured from Sigma Aldrich (UK).

### Organism and maintenance

*A. niger* (wild-type IIB-4) was obtained from IIB Culture Bank, GCU Lahore (Pakistan). The strain was maintained on Potato Dextrose Agar (PDA slants). All the culture media were sterilized in an autoclave (KT-40 L, ALP Co. Ltd., Tokyo, Japan) at 15 lbs/in^2^ for 15 min (121 °C temperature). Fresh slants were inoculated by streaking approximately 10 µL of a dense conidial suspension (10⁶ conidia/mL) from the master culture using a sterile inoculating loop aseptically in a laminar air flow cabinet (Technico Scientific supply, Lahore, Pakistan). The slants were incubated at 30 °C for 3–5 days and preserved at 4 °C in the refrigerator.

### Preparation of inoculum

Ester of sodium sulpho succinic acid (0.05% w/v, 10 mL) was added to a pre-grown slant culture of *A. niger* having profuse growth. The conidial clumps were disrupted with the help of an inoculating wire loop under aseptic conditions. The tube was swirled to form a uniform conidial suspension. A hemocytometer slide was used to determine the conidial count and adjusted to 1.2 × 10^6^ CFU/mL.

### Development of fungal autotrophs

#### Induced mutagenesis by nitrous acid

The wild-type *A. niger* IIB-4 was mutated by nitrous acid for improved citric acid productivity. Nitrous acid solutions (2–12 mM) were formed in potassium phosphate buffer, having pH 7.2. HNO_2_ (1 N 65%) solution and 30% of glycerol solution were prepared. Spore suspension (2 mL each) was added to 2 mL of each nitrous acid solution. The suspensions were kept in shaking incubator for 10 min. After shaking, 1 mL of glycerol solution was added in each mixture. These mixtures were centrifuged for 15 min at 6000 × *g* in a centrifuge machine (Scientific technical corporation Ltd. Lahore, Pakistan). Supernatants were removed and the resulting pellets were washed twice by sterile water. After washing, 2 mL of distilled H_2_O was poured in falcon tubes, containing pellets and then vortex to dissolution factor. Approximately 0.1 mL of these mutated cells was inoculated on six PDA plates, having 0.5% of D-glucose as a substrate. These plates were kept in incubator for three days, at 30 °C. After 3 days, the plates were observed (Justin et al. 2010).

#### De-repression of mutant strain

L-cysteine HCl was used as a selective metabolic inhibitor at concentrations ranging from 0.25 to 1.0 mg/mL, based on internal optimization and methodologies supported by previous studies (Lotfy et al. 2007; Hanif et al. 2004). A 2 mL aliquot of conidial suspension from the mutant strain was mixed with 2 mL of each L-cysteine HCl solution. The mixtures were incubated at 30 °C for 5 min, followed by centrifugation at 4500 × g for 20 min (Scientific Technical Corporation Ltd., Lahore, Pakistan). Supernatants were carefully decanted, and the pellets were washed with sterile distilled water to remove residual L-cysteine HCl.

Mutants that demonstrated survival and continued metabolic activity under these conditions were considered resistant to nitrogen-related repression and were presumed to possess derepressed regulatory traits. The selected L-cysteine HCl-resistant isolates were suspended in acetate buffer (pH 4.5) and stored at 4 °C for further use in productivity evaluations.

#### Screening of resistant auxotrophs

The resistant mutant auxotrophs were screened qualitatively in Petri plates containing nitrogen-deficient Czapek-Dox agar medium, having 30 g/L sucrose, 1 g/L K_2_HPO_4_, 0.5 g/L MgSO_4_.7H_2_O, 0.5 g/L FeSO_4_ and 15 g/L agar with bromocresol green as an indicator. Bromocresol green was added at a final concentration of 0.04% (w/v) in a volume of 1 mL per 100 mL of medium. The medium (approx. 15 mL) was added to sterile Petri plates. After solidification of medium, these plates were inoculated with 0.5 mL of fungal suspension under aseptic environment and were kept in incubator at 30 °C for 3–5 days. Yellow zones appeared around fungal colonies due to citric acid productivity. The best strain of *A. niger* was selected on the basis of wide zones of citric acid in comparison with wild-type and the sub cultured on PDA slants at 30 °C for 3–5 days incubation.

#### Fermentation technique

Continuous culture was employed using 500 mL Erlenmeyer flasks. Hundred millilitres of sucrose mineral salt (SMS) medium at pH 5.5 containing 15 g/L sucrose, 0.25 g/L potassium di hydrogen phosphate, 0.3 g/L ammonium nitrate and 0.03 g/L, was transferred to an Erlenmeyer flask (500 mL). The flask was cotton plugged and autoclaved. After cooling the contents of the flask to an ambient temperature of ~ 20 °C, it was seeded with 1 mL of conidial suspension aseptically. The flask was kept in a shaking incubator (440-DF, Irmeco-GmbH, Germany) at 30 °C (160 rpm) for 7 days (Pandy et al. 2013). The sampling vs. feed was carried out by varying the volumes and time intervals. A repeated-batch culture strategy (often referred to as quasi-continuous) was employed wherein a specific volume of fermented broth was withdrawn and replaced with an equal volume of fresh SMS medium every 24 h following an initial 144-hour fermentation cycle. This method allows for sustained metabolite production while avoiding the complexities of true continuous culture systems.

### Analytical techniques

#### Dry cell weight

Dry weight of mycelium was estimated according to the procedure of Haq and Daud (1995). The culture medium (100 mL) was filtered completely through Whitman’s filter paper (45 μm). The mycelia were thoroughly washed with chilled distilled water and dried at 110 °C for 20 min. The filtrate was utilized for citric acid, protein and sugar analysis.

#### Protein determination

Protein was determined after Bradford (1976) method using bovine serum albumin (BSA) as a standard protein. Bradford reagent (5 mL) was mixed with 0.1 mL of extract. This mixture was kept at room temperature for 5 min. Transmittance was measured at 595 nm through spectrophotometer (UV-1900 Shimadzu, Tokyo, Japan). A control was also run parallel with 0.5 mL of distilled water instead of extract.

#### Citric acid and sugar estimation

Total acid was determined by titrating 10 mL of diluted culture against the standardized 0.1 N NaOH, utilizing phenolphthalein as an indicator. The presence of the light pink colour was the end point (Haq et al. 2004).

$${\rm{\% Total~ acid = }}\frac{{{\rm{Titrant \times Normality~ of ~alkali \times Equivalent~weight~of~acid}}}}{{{\rm{Volume ~of ~sample \times 100}}}}$$

Where total acid is measured in grams per liter (g/L), volume of titrant and sample is measured in milliliters (mL), normality (N) represents per liter of solution (eq/L).

Citric acid in the fermentation broth was determined using a high-performance liquid chromatography (HPLC) system with UV detector (Microsorb-MV, 3.5 mm, 4.15 mm) for acids and reducing sugars (universal column C18) as described earlier (Parvez et al. 1998).

#### Optimization of critical parameters

Different volumes viz. 25, 50, 75, 100, 125, 150 mL of Synthetic Medium for Sporulation (SMS) were employed by the wild-type IIB-4 and mutant strain NA-cys3 of *A. niger* using continuous culture. A range of pH (4–6.5) of SMS medium was also tested. Citric acid fermentation was carried out for 24–192 h under the optimal conditions. Finally, the effect of size of inoculum (2–12%, v/v) was studied.

#### Aconitase co-inhibition by ferrocyanide and methanol

To enhance citric acid productivity, a dual inhibition strategy targeting the aconitase enzyme was applied using potassium ferrocyanide (K₄Fe(CN)₆) and methanol. These inhibitors were added individually and in combination to the fermentation broth at various timepoints. Potassium ferrocyanide was tested at concentrations of 0.01, 0.02, 0.03, and 0.04% (w/v), while methanol was evaluated at 0.5, 1.0, 1.5, and 2.0 mL per 100 mL medium. The compounds were added either at the time of inoculation or at intervals of 2, 4, 8, and 12 h post-inoculation to assess time-dependent effects on aconitase inhibition and citric acid accumulation. The strain NA-CYS3 (mutant) and wild-type IIB-4 were subjected to these treatments under continuous fermentation using SMS medium (pH 4.5) with an inoculum size of 10% (v/v) at 30 °C.

#### Kinetic parameters

Kinetic parameters for batch fermentation were computed after the methods of Lawford and Roseau (1993) and Pirt (1975). The specific growth rate, µ (h^− 1^) was measured from time of incubation vs. ln(X) cultivation plots. Yield coefficients i.e., Y_p/s_ and Y_p/x_ were determined by Y_p/x_ = dP/dX and Y_p/s_ = dP/dS, respectively. The volumetric rates Q_p_ and Q_s_ were estimated from the highest slopes in plots of citric acid productivity and substrate consumption vs. time of incubation. Formation of cell biomass (Q_x_) was assessed from the highest slopes in plots of formation of mycelial cells vs. time of fermentation. Specific rate constants i.e., q_s_ and q_p_ were measured by q_p_ = µ × Y_p/x_ and q_s_ = µ × Y_s/x_, respectively. The specific rate constant q_x_ was assessed by multiplying Y_x/s_ with µ (h^− 1^).

### Statistical analysis

All experiments were conducted in triplicate, and the data were reported as mean ± standard deviation (SD). Treatment effects were analysed by one-way ANOVA (ver.7, SPSS-18). Least protected significance difference method was employed after Snedecor and Cochran (1980). Significant difference among the three replicates was presented as multiple Duncan in the form of probability (*p* < 0.05).

## Results and discussion

### Development of *A. niger* mutant strain by chemical mutagenesis and repression studies

**Fig. 1:**
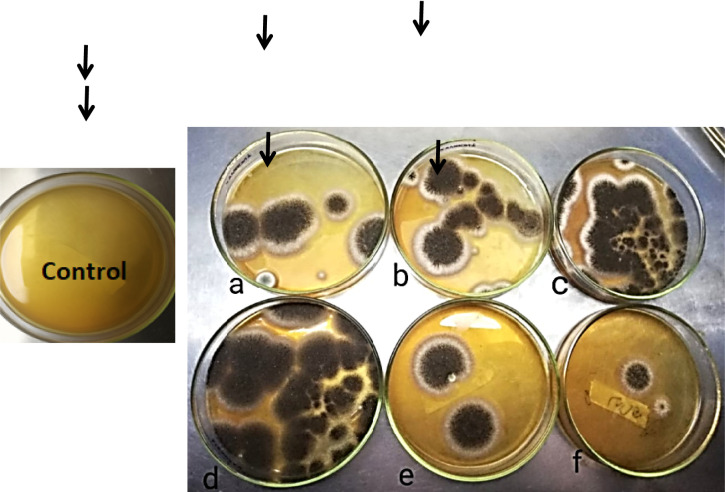
Screening of chemically mutant strains of *A. niger* for a viable nitrogen-requiring auxotroph with enhanced citric acid production potential. Mutants were generated using nitrous acid (2–12 mM) **A** Control plate without fungal inoculation showing no zone of clearance, confirming the absence of background hydrolysis or spontaneous clearing in the medium. **B** Fungal-inoculated plate showing visible zones of glucose hydrolysis suggesting extracellular enzymatic activity by the fungal strain. Both plates were incubated under identical conditions

The induced mutagenesis of wild-type *A. niger* IIB-4 by HNO_2_ (2–12 mM) was accomplished for increased citric acid productivity. The results are given in Table 1. The wild-type produced 2.35 g/L of citric acid. In Fig. 1 is depicted the screening of mutant strains of *A. niger* for a viable nitrogen-requiring auxotroph bearing citric acid potential. The individual plates a, b, c, d, e and f exhibited clear zones of glucose-hydrolysis. The variants were picked up and subjected to batch fermentation under optimal conditions viz. volume of SMS media (25 mL), initial pH (3.5), time of incubation (168 h) and inoculum size (4%, v/v). When suspension culture was exposed to 2 mM the death rate was only 60%. Among all variants, NA-10 strain was selected for maximum citric acid productivity. NA-10 strain, mutated by 10 mM concentration of nitrous acid, resulted in 4.16 g/L of citric acid that was 1.8 times more concentration than the wild-type with a concomitant better sugar consumption rate. It might be due to the fact that HNO_2_ is a strong mutagenic agent, which can induce permanent changes in DNA structure as reported by Justin et al. (2010).

**Table 1:** Nitrous acid (NA) induced mutagenesis of *Aspergillus niger* and development of a viable nitrogen-requiring mutant auxotroph for enhanced citric acid productivity in batch culture*

Mutagenesis	Death rate (%)	No. of survivor colonies	Strain coding	Citric acid (g/L)
Wild-type	-	-	IIB-4	2.35 ± 0.16
NA (mM)				
2	60	3	NA-1 - NA-3	2.48–3.18
4	70	2	NA-4, NA-5	3.24–3.45
6	80	2	NA-6, NA-7	3.53–3.74
8	85	2	NA-8, NA-9	3.77–4.12
10	90	1	NA-10	4.16 ± 0.218
12	95	1	NA-11	3.49 ± 0.174
L-Cysteine HCl (mg/L)				
0.25	90	2	NA-cys1, NA-cys2	4.05–4.11
0.5	95	1	NA-cys3	4.32 ± 0.216
0.75	95	1	NA-cys4	3.85 ± 0.192
1	95	1	NA-cys5	3.28 ± 0.164

*SMS volume 25 ml, initial pH 3.5, time of incubation 168 h, inoculum size 4% (v/v)

The strain NA-10 was subjected to different concentrations (0.25–1 mg/L) of L-cysteine HCl to prevent reversion. When it was exposed to 0.25 mg/L, 90% death rate occurred and the range of citric acid was 4.05–4.11 g/L. The putative mutant NA-cys3 thrived at 0.5 mg/L of L-cysteine HCl with 95% death rate. The maximum citric acid productivity by NA-cys3 was 4.32 g/L that was even higher than that of NA-10. Although the chemical mutagenesis and auxotroph screening steps effectively led to the isolation of a high-yielding L-cysteine HCl-resistant mutant strain (NA-cys3), this study did not include genetic validation of the induced mutations. Whole-genome sequencing or PCR-based confirmation of mutation sites was not performed due to resource limitations. Nonetheless, the observable phenotypic changes, namely, L-cysteine HCl resistance and significantly enhanced citric acid production, serve as strong indicators of successful mutagenesis. Future research should incorporate genome sequencing, transcriptomic profiling, or site-specific mutation analysis to identify genetic determinants responsible for improved metabolic performance. Such characterization will facilitate deeper insights into the pathways affected by nitrous acid mutagenesis and may inform further rational strain engineering.

### Optimization of size of inoculum and incubation period

**Fig. 2:**
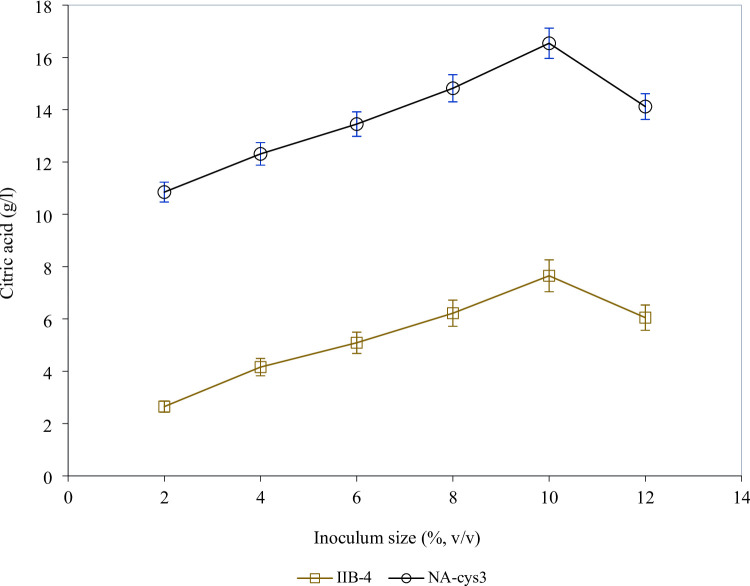
Effect of different inoculum sizes (2–12%) on citric acid productivity by wild-type strain IIB-4 and auxotrophic mutant NA-CYS3. Fermentation was carried out in 50 mL SMS medium at initial pH 4.5, incubated for 144 h. Error bars represent standard deviation (5%) among triplicate experiments

In Fig. 2, the effect of size of inoculum on citric acid fermentation was examined by using various percentage of inoculum such as 2–12% (v/v). Using 2% inoculum, citric acid productivity by wild-type and mutant strain was 2.65 g/L and 10.85 g/L, respectively. The yield of citric acid was less might be due to the reason that at low inoculum concentration, enough mycelia was not formed to convert more sugars into citric acid. The mutant strain produced 4-fold higher concentration than wild-type. Citric acid productivity was increased with increase in inoculum size. Maximum amount of citric acid (500 mL) was produced at 10% (v/v) inoculum. With this inoculum size, the wild-type produced 7.65 g/L of citric acid while the mutant strain produced 16.54 g/L of citric acid that was 2.1-fold high concentration than wild-type. However, further increase in size of inoculum led to decrease citric acid yield. Because the fermentation medium became more viscous that lead to the diffusion problems. It is clear that citric acid productivity is less (4.16 g/L by wild-type; 12.31 g/L by mutant strain) when the inoculum size was 4% (v/v). Consequently, 10% (v/v) size of inoculum was optimized for citric acid batch culture. Similar study was conducted by Bari et al. (2009), who produced maximum citric acid from palm fruit, with 10% inoculum concentration. Ali et al. (2002) obtained maximum citric acid productivity with 1% of inoculum size. Based on literature, the possible reasons could be strain variation, medium composition, oxygen transfer and viscosity, fermentation conditions, fungal morphology, and mutant vs. wild type performance (Papagianni et al. 2004).

**Fig. 3:**
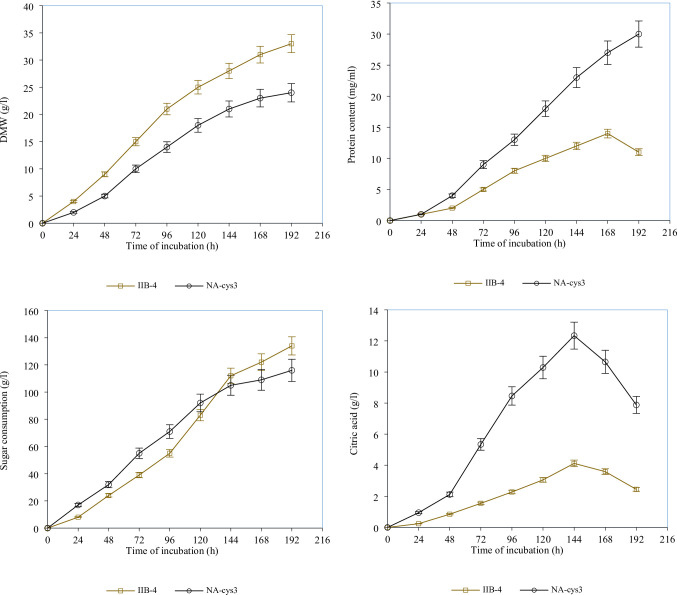
Effect of incubation time (24–192 h) on dry biomass, protein content, sugar consumption, and citric acid production by *A. niger* strains IIB-4 and NA-CYS3. Conditions: SMS 50 mL, initial pH 4.5, inoculum size 4% (v/v). Data are means ± SD and error bars represent standard deviation (5%) among triplicate experiments

The incubation time period of fermentation medium was varied from 24 to 192 h. Dry mycelial biomass, protein content, sugar consumption and citric acid productivity were examined with these various fermentation periods as shown in Fig. 3. At 24 and 48 h of incubation the productivity of citric acid was very less but then it gradually increased up to 144 h of fermentation. The minimal citric acid productivity was observed (0.24 g/L) for IIB-4 and (0.95 g/L) for NAcys3 at 24 h of fermentation time period. The mutant strain produced 2.9-fold higher concentration of citric acid (12.34 g/L) than concentration produced by wild-type (4.12 g/L). Dry mycelial biomass, protein content and consumed sugar content for wild-type were 28 g/L, 12 mg/mL and 112 g/L, respectively. For NA-cys3, the dry mass was 21 g/L, protein content was 23 mg/L and consumed sugar content was 105 g/L. Beyond 144 h of incubation, a decline in citric acid productivity was observed. It might be due to fungal age, production of inhibitors by *A. niger*, decay in enzyme system responsible for citric acid productivity, reduction in sugar content of the medium and available nitrogen (Lofty et al. 2007). The results were also supported by Watanabe et al. (2009), who performed fermentation process of citric acid for 72–216 h using medium with 150 g/L of sugar content. They achieved 68.2% yield of citric acid by consuming the provided reducing sugars.

**Fig. 4:**
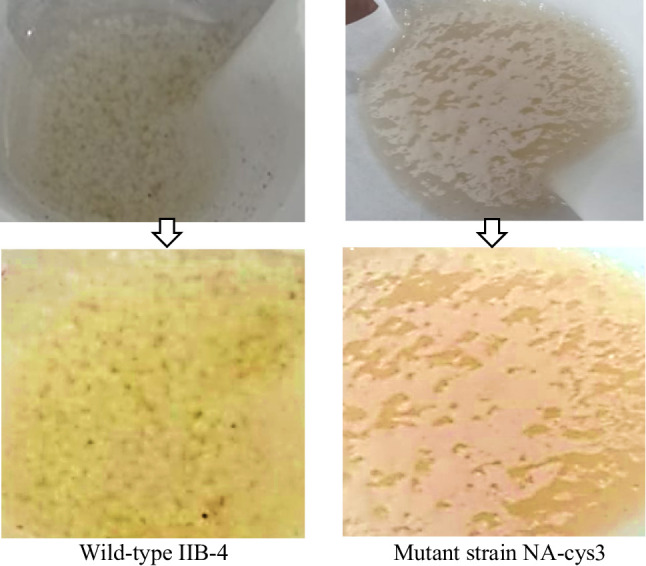
Morphological comparison of pre-grown mycelia (one zoomed-in and one zoomed-out) of wild-type IIB-4 and mutant strain of *A. niger* NA-CYS3 under optimal conditions

Morphological comparison of pre-grown mycelia of wild-type IIB-4 and mutant strain of *A. niger* NA-cys3 under optimal conditions was made (Fig. 4). NA-cys3 has superior morphological forms of pellets with highly organized mycelial pattern in comparison to IIB-4. This can be attributed to hyper citric acid productivity as reported by Zheng et al. (2022). Pirt (1975) reported the similar result that variant strain of *A. niger* expressed better ability for sugar consumption with a faster growth rate which led to enhanced yield of citric acid. Zhang et al. (2019) obtained higher citric acid yield from mutant strain of *A. niger* than wild-type.

### Kinetic studies for citric acid productivity

**Fig. 5:**
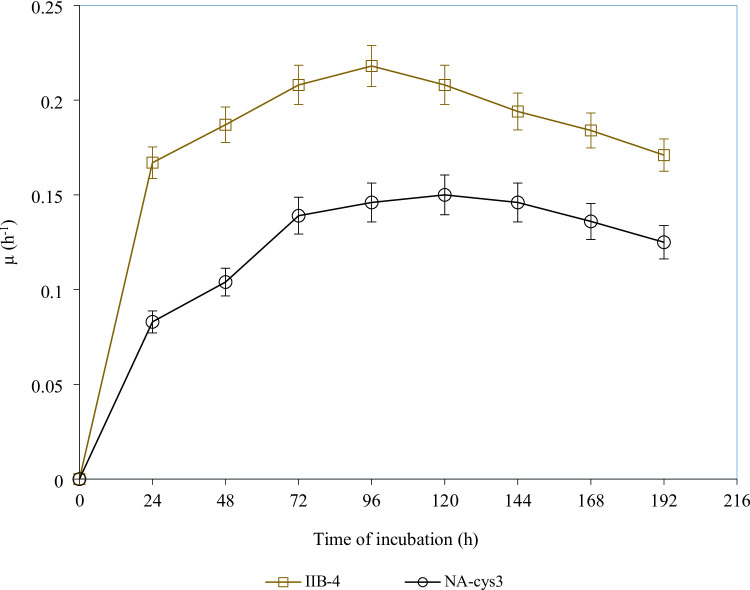
Comparison of specific growth rate (h^–1^) for citric acid productivity by wild-type IIB-4 and auxotrophic mutant strain NA-cys3 of *A. niger*. Conditions: SMS 50 ml, pH 4.5, inoculum size 4% (v/v). Error bars indicate standard deviation (SD set at 5%) among three parallel replicates

The specific growth rate µ (h^− 1^) was determined and the results are shown in Fig. 5. After 24 h of fermentation period, the specific growth rate by wild-type was 0.167 h^− 1^ and by variant strain was 0.083 h^− 1^. The highest value of specific rate of growth (0.15 h^− 1^) was however shown after 120 h of incubation by mutant strain but maximum citric acid productivity was achieved after 144 h of fermentation. At 144 h of incubation, the wild-type showed (0.194 h^− 1^) specific growth rate and mutant strain showed (0.146 h^− 1^) specific growth rate. After 144 h, the specific growth rate of both wild-type and mutant strain decreased with increased in time period. The results were according to the findings of Ali et al. (2002).

**Fig. 6:**
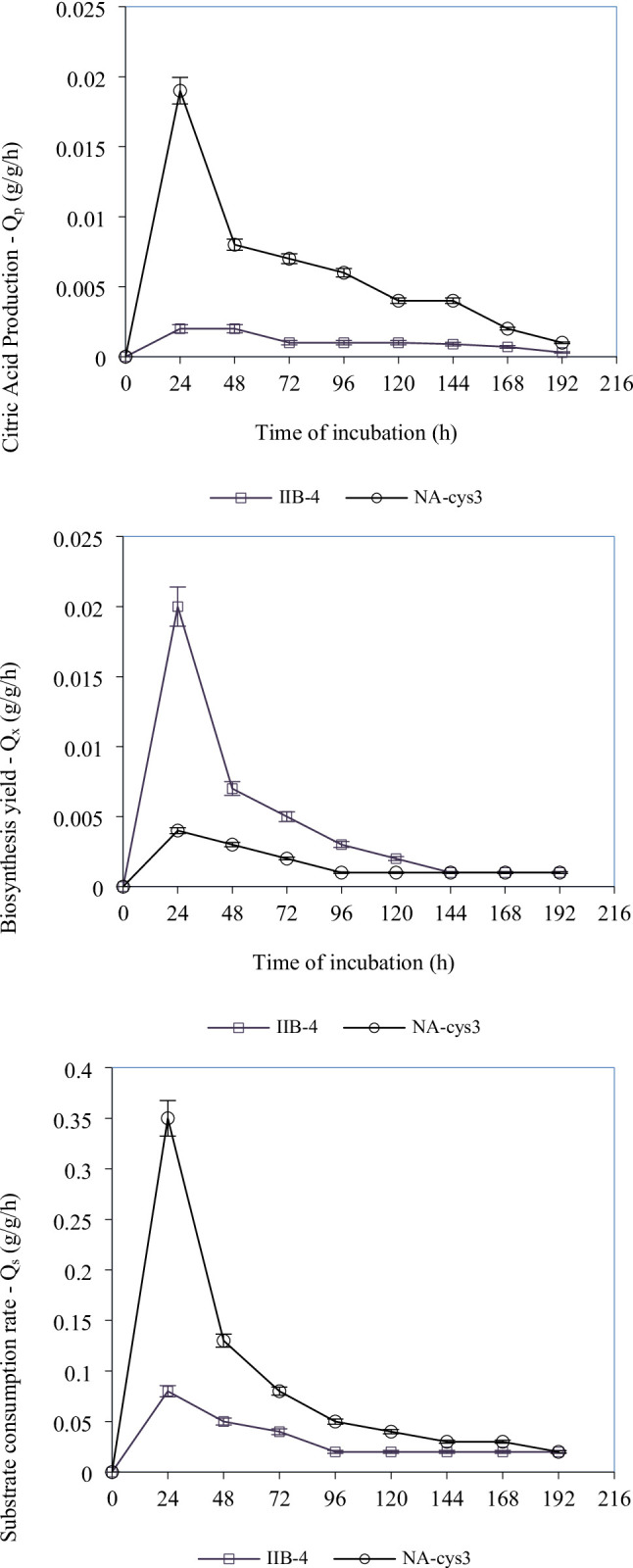
Comparison of volumetric rates for citric acid productivity by wild-type IIB-4 and auxotrophic mutant strain NA-cys3 of *A. niger*. Volumetric rate constant for product formation = Q_p_ (g/g/h), volumetric rate constant for dry cell weight formation = Q_x_ (g/g/h), volumetric rate constant for substrate consumption = Q_s_ (g/g/h). Inhibitor-free batch culture conditions: 50 mL SMS, pH 4.5. Error bars show mean ± SD for three replicates; significant differences noted at *p* ≤ 0.05 using one-way ANOVA

The data in Fig. 6 showed volumetric rates like Q_p_, Q_s_ and Q_x_ under various fermentation periods 12–192 h. The comparison of Q_p_ (g/g/h) showed that the value of it was highest for first 24 h but then gradually decreased with increasing time of incubation. It was found to be 0.002 g/g.h for wild-type and 0.019 g/g.h for variant strain at 24 h of cultivation. With 144 h of fermentation the wild-type showed 0.0009 g/g.h value of Q_p_ and mutant strain had 0.004 g/g h value of Q_p_. The mutant showed 4.4-fold higher value of Q_p_ than wild-type. In this research, the highest value for Q_p_ is several times enhanced over the findings of Rohr et al. (1987). This enhancement can be attributed to the effective aconitase co-inhibition strategy, which redirected the metabolic flux towards citric acid accumulation, while the observed reduction in the volumetric rate constant for dry mycelial mass (Qx) indicates a shift from biomass production to product formation, further supporting higher citric acid productivity (Papagianni 2007). It was observed that the volumetric rate constant for dry mycelial mass (Q_x_) was reduced with rise in fermentation period for both wild-type and variant strain. The highest value was measured (0.02 g/g h) for wild-type and (0.004 g/g h) for variant strain at 24 h of fermentation.

After 144 h of incubation the values for Q_x_ with wild-type and mutant strain were noted, that were equal to each other (0.001 g/g h). The analysis of volumetric rate Q_s_ (g/g h) for substrate utilization revealed that mutants are more capable of consuming substrate than wild-type. It is due to the enhanced metabolic pathways, reduced feedback inhibition, increased transporter expression, improved stress tolerance, and redirected metabolic flux towards product formation (Papagianni 2007). The value of volumetric rate constant for substrate consumption was highest for 24 h of incubation, the wild-type showed 0.08 g/g h Q_s_ and the mutant strain expressed 0.35 g/g h of Q_s_. Beyond 24 h of fermentation period, the value of Q_s_ was reduced with further increase in fermentation period (up to 192 h).

**Fig. 7:**
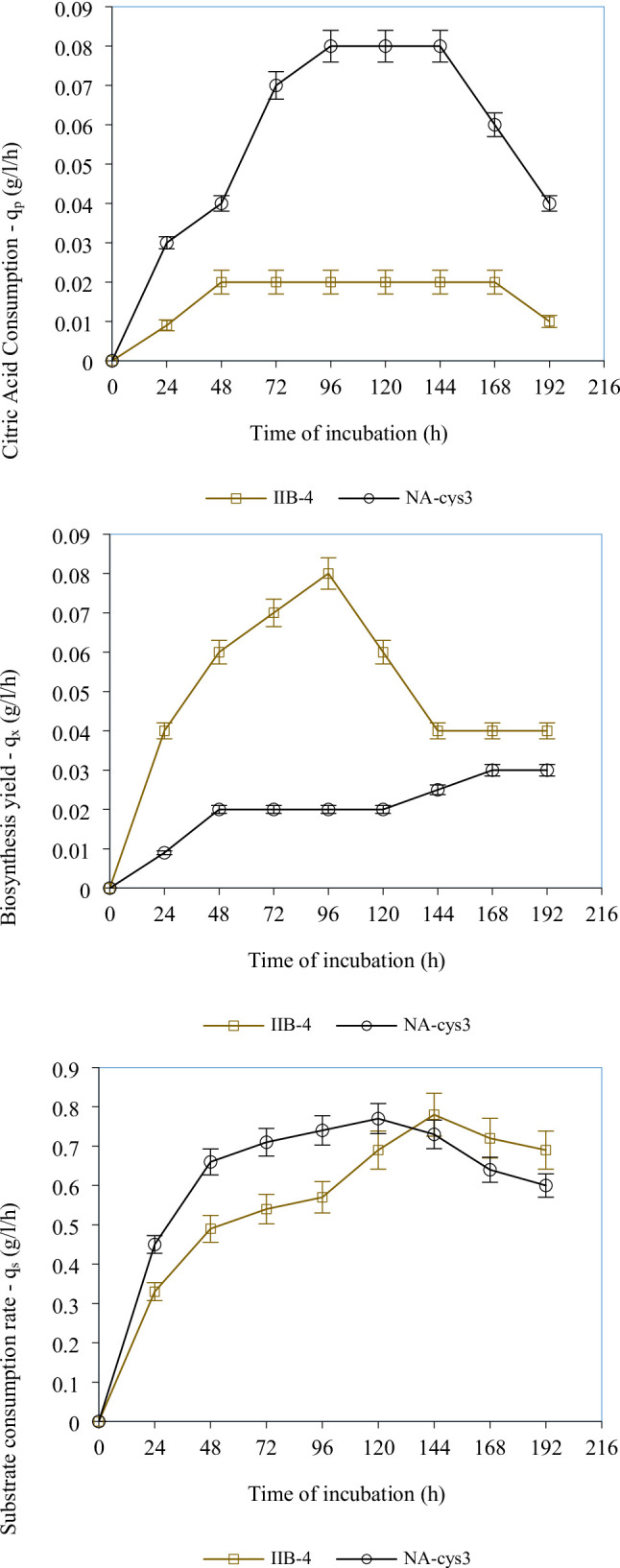
Comparison of specific rate constants for citric acid productivity by wild-type IIB-4 and auxotrophic mutant strain NA-cys3 of *A. niger*. Sp. rate constant for product formation = q_p_ (g/L/h), sp. rate constant for dry cell weight = q_x_ (g/L/h), sp. rate constant for substrate consumption = q_s_ (g/L/h). Fermentation conditions: pH 4.5, inoculum 10% (v/v). Data (mean ± SD) are based on triplicate experiments; significant differences noted at *p* ≤ 0.05 using one-way ANOVA

Specific rate constants like q_p_, q_s_ and q_x_ were examined and assessed for IIB-4 and NA-cys3 strain of *A. niger* under various time periods of fermentation (24–192 h) as highlighted in Fig. 7. Specific rate constant for product synthesis (q_p_) was (0.009 g/L/h) for IIB-4 and (0.03 g/L/h) for variant strain at 24 h of cultivation. It was noted that the value of q_p_ enhanced with increase in fermentation time period. The value increased due to the metabolic shift from biomass production to product formation, leading to higher citric acid accumulation as aconitase activity was inhibited. A highest value was obtained (0.02 g/L/h) for IIB-4 at 72–168 h and then it was reduced at 96 h whereas, its value was maximum (0.08 g/L/h) for NA-cys3 at 96–144 h of fermentation and then declined with further increase of time period. The mutant strain showed 4 times increased value of q_p_ than IIB-9 isolate. The results of q_p_ were several times better than the cultures studied by previous workers (Pirt 1975). Specific rate constant for dry mycelial mass (q_x_) was (0.08 g/L/h) for wild-type and (0.009 g/L/h) for variant strain at 24 h of fermentation period.

At 144 h of incubation the q_x_ was 0.04 g/L/h and 0.02 g/L/h for wild-type and mutant strain, respectively. Specific rate constant for substrate consumption, q_s_ was (0.33 g/L/h) for wild-type and (0.70 g/L/h) for mutant strain at 24 h of fermentation. The variant strain expressed 2.1 times greater value of q_s_ than wild-type. It was due to due to enhanced substrate uptake, likely resulting from metabolic modifications that increased transporter activity or reduced feedback inhibition. It was observed to be maximum (0.78 g/L/h) for wild-type at 144 h of fermentation period and then its value was decreased with rise in time period. For NA-cys3, the q_s_ was highest (0.77 g/L/h) at 120 h of fermentation period.

**Fig. 8:**
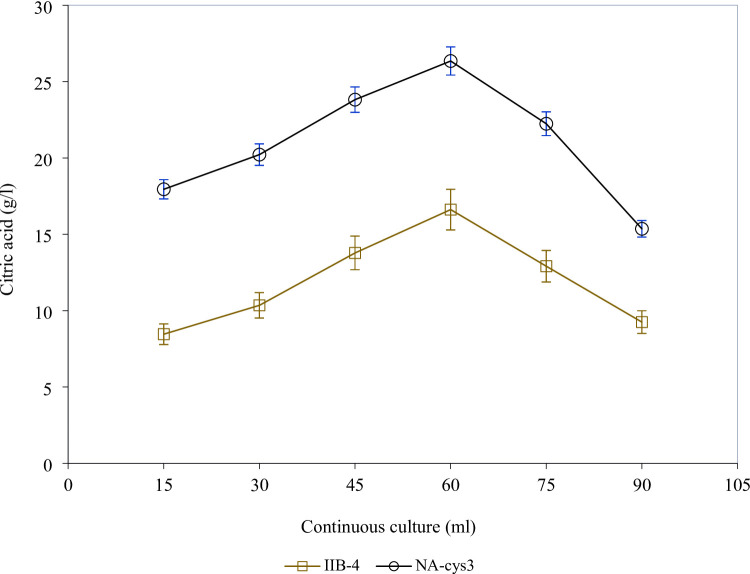
Continuous citric acid productivity by wild-type IIB-4 and auxotrophic mutant strain NA-cys3 of *A. niger*. Initial pH 4.5, time of incubation 24 h for each cycle, inoculum size 10% (v/v). Bars indicate standard deviation (SD set at 5%) among three parallel replicates. The sum means values vary significantly at *p* ≤ 0.05 from each other under one-way ANOVA

After optimizing all parameters in batch fermentation, the wild-type and mutant strain of *A. niger* were processed to continuous fermentation. The data of Fig. 8 showed citric acid productivity in continuous culture of *A. niger*. Following an initial 144-hour fermentation, a repeated-batch fermentation strategy was employed in which varying volumes of fermented broth (15–60 mL) were replaced every 24 h with fresh SMS medium. This approach mimics continuous operation but without constant flow, and is thus referred to as repeated-batch fermentation. When 15 mL of fermented broth was replaced by 15 mL of fresh SMS media, fermented broth was subjected to citric acid analysis. The IIB-4 strain produced 8.46 g/L of citric acid and 17.95 g/L of citric acid was obtained by mutant strain. With increase in volume, the enhancement in citric acid productivity was observed. The highest productivity was obtained when 60 mL of fermented broth was replaced with 60 mL of fresh medium. The wild-type resulted in 16.62 g/L of citric acid and mutant strain produced 26.35 g/L of citric acid. The mutant strain showed 1.6-fold higher production than wild-type. Further increment in volume resulted in decreased citric acid productivity. Kim et al. (2002) reported the continuous and batch fermentation of an immobilized *A. niger*. The citric acid yield obtained by continuous fermentation was 1.3-fold higher than batch fermentation. Similar results were reported by Gupta and Sharma (1994), who used the cultures continuously for 30 days without any apparent decrease in citric acid productivity.

### Biochemical co-inhibition of aconitase in continuous culture

The application of a biochemical aconitase co-inhibition strategy was evaluated to redirect the carbon flux from isocitrate toward citric acid accumulation. Potassium ferrocyanide, a known potential inhibitor of iron-sulfur cluster-containing enzyme like aconitase, was selectively used to inhibit the conversion of citrate to isocitrate. This blockage effectively led to a metabolic bottleneck that favoured citrate accumulation in the extracellular medium. In parallel, methanol was employed as a co-inhibitor due to its ability to permeabilize fungal membranes and possibly induce oxidative stress, both of which are known to enhance the secretion and biosynthesis of organic acids, including citric acid.

**Fig. 9:**
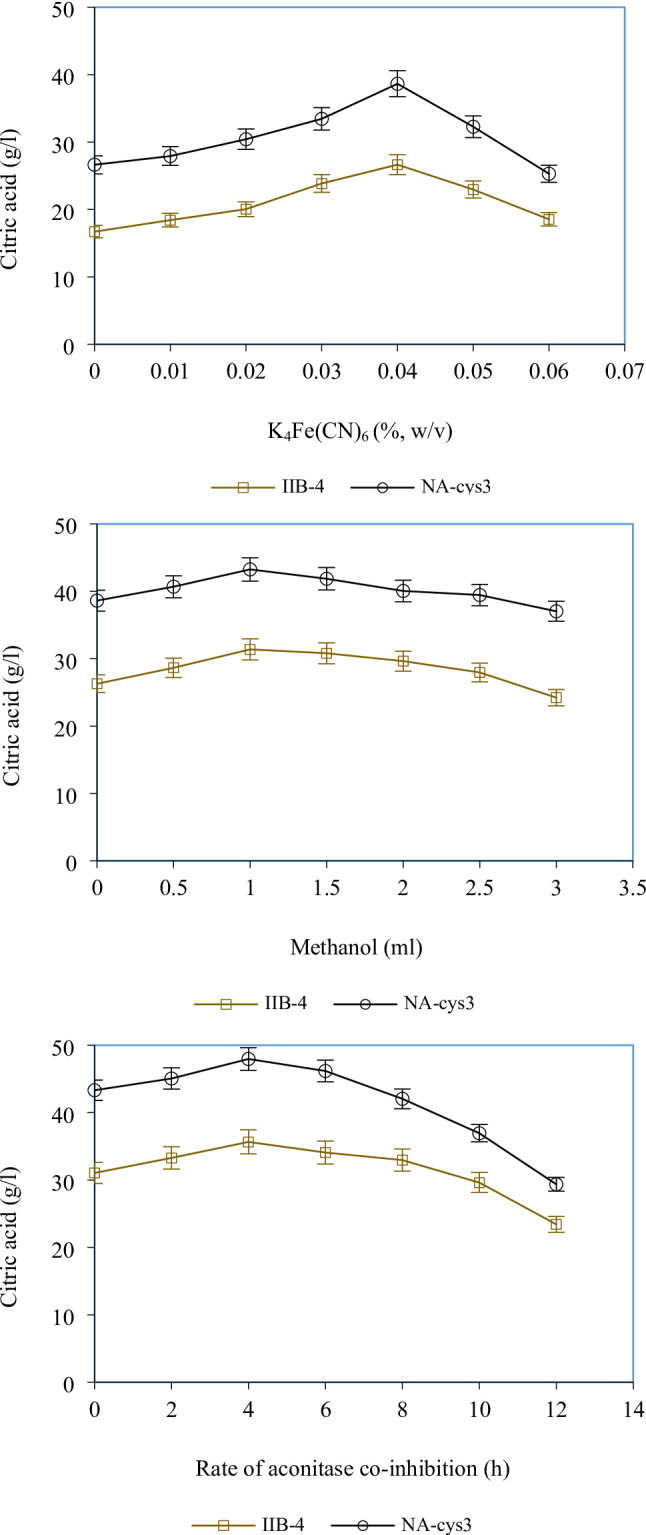
Effect of aconitase co-inhibition using K_4_Fe(CN)_6_ (0.004%) and methanol (1 mL) on continuous citric acid productivity by wild-type and mutant NA-cys3 strains of *A. niger*. Individual panels: **a** K_4_Fe(CN)_6_, **b** methanol, **c** Overall rate of fermentation. Conditions: pH 4.5, time of incubation 24 h, inoculum size 10% (v/v). Bars indicate standard deviation (SD set at 5%) among three parallel replicates. The sum means values vary at *p* ≤ 0.05 from each other under one-way ANOVA

The effect of various concentration of K_4_Fe(CN)_6_ on continuous citric acid fermentation by *A. niger* was tested as shown in Fig. 9a. K_4_Fe(CN)_6_ was added at inoculation time. When there was no K_4_Fe(CN)_6_ in medium, citric acid productivity by wild-type and mutant strain was 16.71 g/L and 26.64 g/L, respectively. Citric acid productivity increased with increase in concentration of potassium ferrocyanide. At 0.04% (w/v) citric acid productivity was maximum for both wild-type and mutant strain. The wild-type produced 26.65 g/L and mutant strain produced 38.65 g/L of citric acid. In this study, citric acid productivity was due to the inhibition of aconitase by the iron from the potassium ferrocyanide, added in the medium. Same study was also demonstrated by El-Hussein et al. (2009), in which enhanced citric acid productivity and its yield was obtained with addition of ferrocyanide in medium. Further increment in potassium ferrocyanide concentration led to decline in productivity of citric acid probably due to the decreased sugar consumption and toxic effects of potassium ferrocyanide on growth of fungal mycelium.

Methanol has stimulatory effect on citric acid productivity in process of fermentation (Ali et al. 2002). The data in Fig. 9b highlighted the effect of methanol on inhibition of aconitase for continuous citric acid productivity by *A. niger*. Different concentrations of methanol were added at time of inoculation along with 0.04% (w/v) of potassium ferrocyanide. At 0 mL concentration of methanol, citric acid productivity by wild-type was 26.28 g/L and by mutant strain was 38.62 g/L. When methanol’s concentration was increased up to 1 mL, maximum productivity of citric acid was achieved. The IIB-4 strain produced 31.38 g/L of citric acid and NA-cys3 resulted in 43.25 g/L of citric acid. The mutant showed 1.3 times more citric acid productivity than wild-type. It might be due to that it enhanced permeability of cell membrane that led to better secretion of citric acid from fungal cells (Papagianni 2007; Ruijter et al. 2000).

Moreover, it increased the metabolic activity of citric acid synthase and the uptake and assimilation of carbon source (Yu et al. 2017). Beyond 1 mL concentration, decline in citric acid productivity was observed by both wild-type and mutant strain. The poor productivity by both wild-type (24.22 g/L) and mutant strain (37.03 g/L) was observed when 3 mL of methanol was added in the medium. It might be due to the disturbance in inoculum morphology and fungal metabolism (Navaratnam et al. 1998; Haq et al. 2003). Adudu et al. (2019) achieved maximum citric acid yield at low concentration of methanol. Nadeem et al. (2010) found 1.5% methanol as optimal level for maximum citric acid productivity.

The optimized concentrations of potassium ferrocyanide (0.04%, w/v) and methanol (1 mL) were added together at different rates after inoculation as displayed in Fig. 9c. The productivity by wild-type and mutant strain was 31.05 g/L and 43.32 g/L, respectively. Less production was achieved because when they were added at the time of inoculation potassium ferrocyanide drastically disturbed the mycelial growth. The maximum production was achieved when potassium ferrocyanide and methanol were added after 4 h of inoculation. The IIB-4 produced 35.65 g/L of citric acid and NA-cys3 resulted in 48.96 g/L citric acid productivity. Nadeem et al. (2010) examined that citric acid productivity increased with increase in time for addition of methanol. The mutant strain showed 1.3 times more citric acid productivity than wild-type. This may be due to genetic modifications that enhanced citrate synthase activity, reduced aconitase activity, improved substrate utilization, or increased cell membrane permeability for better citric acid secretion. The citric acid productivity decreased with further increase in time course of addition of methanol and ferrocyanide. The least production by wild-type (23.42 g/L) and mutant strain (29.36 g/L) was obtained when methanol and ferrocyanide were added after 12 h of addition of inoculum. Haq et al. (2003) demonstrated that highest productivity of citric acid was attained with addition methanol in to the fermentation medium at different time of incubation, but with further increase in time period of incubation led to decrease in citric acid productivity.

Despite the substantial increase in citric acid productivity observed through biochemical aconitase co-inhibition using potassium ferrocyanide and methanol, the absence of a genetically engineered aconitase-deficient control strain limits the mechanistic resolution of our findings. While ferrocyanide is known to inhibit iron–sulfur cluster-containing enzymes such as aconitase, and methanol can modulate central metabolic fluxes by inducing oxidative stress and membrane permeability, these effects were inferred rather than directly validated in this study. A genetically confirmed aconitase knockdown or knockout strain would serve as a more definitive negative control to isolate the specific impact of aconitase inhibition. Additionally, direct measurement of aconitase and citrate synthase enzymatic activities, as well as transcriptional profiling of corresponding genes, would be necessary to verify the metabolic shift hypothesized to enhance citrate accumulation. Such molecular-level analyses were beyond the scope of the present work but are planned for future investigations. These strategies would greatly strengthen the mechanistic understanding of the dual inhibition approach and confirm the role of aconitase suppression in redirecting carbon flux toward citric acid biosynthesis.

Moreover, while the present study employed a classical one-variable-at-a-time (OVAT) approach to evaluate the effects of individual parameters, future optimization efforts will benefit from statistically guided models. In particular, the use of Response Surface Methodology (RSM) or Design of Experiments (DoE) can reduce the number of required experimental trials and uncover interactive effects among key variables such as inoculum size, incubation time, and inhibitor concentrations. These approaches are planned for the next phase of process optimization to further enhance citric acid yield and production efficiency.

### Kinetic evaluation of ferrocyanide and methanol-based aconitase co-inhibition

The data of Table 2 showed the whole evaluation of kinetic parameters for aconitase co-inhibition of continuous citric acid productivity by wild-type and variant strain of *A. niger*. The specific growth showed by wild-type and mutant strain was (0.41 h^− 1^) and (0.19 h^− 1^), respectively. The value of yield coefficient for product synthesis on the basis of dry mycelial mass Y_p/x_, obtained by wild-type was 0.34 g/g/L and variant strain was 0.95 g/g/L that were 2.7-fold higher than wild-type. The product yield Y_p/s_ showed by wild-type was 0.12 g/g/L and by variant strain was 0.39 g/g/L, that was 3.2 times higher than wild-type. The volumetric rate constant for product formation Q_p_, expressed by wild-type was (0.002 g/g/h) and by mutant strain was (0.008 g/g/h). The mutant strain showed 4 times increase in value than wild-type. The volumetric rate Q_x_, obtained by mutant strain (0.007 g/g/h) was 2.3-fold higher than wild-type (0.003 g/g/h). The Specific rate qp for NA-cys3 (0.17 g/L/h) was 1.4-fold higher than IIB-4 (0.12 g/L/h). The specific rate for dry cell weight formation q_x_, showed by wild-type was 0.18 g/L/h and by mutant strain were 0.27 g/L/h, which was 1.5-fold higher than wild-type. To provide a consolidated comparison of performance metrics, Table 3 summarizes citric acid yield, yield coefficient (Yp/s), and volumetric productivity (Qp) for both the wild-type and NA-CYS3 mutant strains under all tested fermentation conditions.

**Table 2:** Overall comparison of kinetic parameters for aconitase co-inhibition of continuous citric acid productivity by wild-type IIB-4 and auxotrophic mutant strain of A. niger*

Kinetic variables	Kinetic quotients	Units	Kinetic models	Modes	Comparative values	
					IIB-4	NA-CYS3
Specific growth	µ	h^− 1^	Growth rate	Cellular formation	0.41 ± 0.12	0.19 ± 0.16
Product yield	Y_p/x_	g/g/L	Yield coefficient	Metabolic production	0.34 ± 0.11	0.95 ± 0.15
Product yield	Y_p/s_	g/g/L	Yield coefficient	Metabolic production	0.12 ± 0.004	0.39 ± 0.13
Volumetric rate	Q_p_	g/g/h	Rate constant	HA Production	0.002 ± 0.001	0.008 ± 0.002
Volumetric rate	Q_x_	g/g/h	Rate constant	Cellular formation	0.003 ± 0.001	0.007 ± 0.002
Specific rate	q_p_	g/L/h	Rate	HA Production	0.12 ± 0.01	0.17 ± 0.02
Specific rate	q_x_	g/L/h	Rate	Cellular formation	0.18 ± 0.04	0.27 ± 0.03

Specific growth rate = µ (h^− 1^), Yield coefficient for product formation on the basis of dry cell weight formation = Y_p/x_ (µg/g/L), Yield coefficient for product formation on the basis of substrate consumption = Y_p/s_ (µg/g/L), Volumetric rate constant for product formation = Q_p_ (µg/g/h), Volumetric rate constant for dry cell weight formation = Q_x_ (µg/g/h), Specific rate constant for product formation = q_p_ (µg/L/h), Specific rate constant for dry cell weight formation = q_x_ (µg/L/h)

±Indicate standard deviation amongst the values of three parallel replicates

**Table 3:** Summary of citric acid production, yield coefficients (Yp/s), and volumetric productivity (Qp) for wild-type and NA-CYS3 strains under various tested fermentation conditions. Data represent mean values from triplicate experiments

Condition	Strain	Citric Acid Yield (g/L)	Yp/s (g/g)	Qp (g/g/h)
Batch (no inhibitor)	Wild-type (IIB-4)	2.35	0.12	0.002
Batch (no inhibitor)	NA-CYS3	4.32	0.39	0.008
Continuous (no inhibitor)	Wild-type (IIB-4)	16.62	0.12	0.002
Continuous (no inhibitor)	NA-CYS3	26.35	0.39	0.008
Continuous (*K₄Fe(CN)₆* + Methanol)	Wild-type (IIB-4)	35.65	0.12	0.002
Continuous (*K₄Fe(CN)₆* + Methanol)	NA-CYS3	48.96	0.39	0.008

While the biochemical co-inhibition strategy demonstrated promising increases in citric acid productivity at the laboratory scale, it is important to acknowledge that a comprehensive cost–benefit analysis at an industrial scale is necessary to validate the economic feasibility of introducing enzyme inhibitors into the fermentation medium. Such analysis should consider not only the cost of inhibitors but also potential impacts on process complexity, safety, downstream processing, and regulatory compliance. Moreover, for food-grade citric acid production, potassium ferrocyanide must be removed during downstream processing to meet safety standards. Alternative non-toxic aconitase inhibitors or food-grade extraction techniques may be explored in future work.

## Conclusion and prospects

This study presents a dual-strategy approach to improve citric acid production in *A. niger* by combining chemical mutagenesis and biochemical aconitase co-inhibition. A novel mutant strain, NA-CYS3, was successfully developed using nitrous acid and further screened for resistance to L-cysteine HCl. Notably, the transition from batch to continuous fermentation allowed for a considerable reduction in production time, achieving target yields within 24 h compared to the conventional 144-hour process. Under optimized batch and continuous fermentation conditions, NA-CYS3 consistently outperformed the wild-type strain in terms of citric acid yield and kinetic efficiency. The use of potassium ferrocyanide and methanol as co-inhibitors effectively enhanced citrate accumulation by selectively targeting aconitase activity. When both inhibitors were added four hours post-inoculation, the mutant strain achieved a maximum citric acid concentration of 48.96 g/L, a 1.3-fold increase over the wild-type. Kinetic analysis confirmed significant improvements in substrate utilization (Yp/s), product yield (Yp/x), and volumetric productivity (Qp).

While the outcomes support the effectiveness of this dual approach, several limitations remain. Genetic validation of the mutant strain was not conducted, and enzyme activity assays for aconitase and citrate synthase were not performed. There is also no clear negative control for aconitase inhibition, such as a condition combining methanol and ferrocyanide in a strain with genetically knocked-down or inactivated aconitase. Furthermore, long-term strain stability, by-product profiling, and potential ferrocyanide toxicity at industrial scale require further investigation. Future studies should include genome sequencing and transcriptomic profiling to identify mutation sites and confirm the mechanistic roles of targeted enzymes. Enzymatic assays or gene expression analyses for aconitase and citrate synthase activity would provide critical support for the proposed mechanism. Additionally, pilot-scale trials, coupled with omics-based insights, will help assess the feasibility and robustness of this strategy for commercial application. Overall, the findings demonstrate a promising framework for cost-effective, scalable citric acid production through strain engineering and metabolic control.

## Supplementary Information

Below is the link to the electronic supplementary material.

Supplementary Material 1

## Data Availability

Data will be made available on request.
