# Supplementary material for: Enhanced citric acid production via aconitase co-inhibition strategy using L-cysteine HCl-resistant Aspergillus niger NA-CYS3
Source: 3 Biotech. 2026 Jun 27;16(7):290. doi: 10.1007/s13205-026-04929-2 (PMC13310209; doi:10.1007/s13205-026-04929-2)
Supplement: Supplementary file 1 — Supplementary Material 1 [file 13205_2026_4929_MOESM1_ESM.docx]

**Authors’ Response to Reviewer #1:**

**1. Novelty and Significance**

**Strength: The dual strategy of chemical mutagenesis and aconitase co-inhibition is a valuable contribution to improving citric acid yields.**

**Suggestion: Clarify in the introduction how this strategy differs from previous methods—what exactly is novel here? Is it the co-inhibition approach, the mutant selection strategy, or the optimization design?**

**Response:** We thank the reviewer for the comment. In the revised manuscript, we have clarified in the Introduction (**Lines 66-75**) that the novelty lies in the dual strategy—a combination of chemical mutagenesis to create an L-cysteine HCl-resistant *A. niger* mutant (NA-CYS3) and the co-inhibition of aconitase enzyme using potassium ferrocyanide and methanol. While individual strategies such as mutagenesis or methanol supplementation have been previously reported (**Lines 46-65,** e.g., Papagianni, 2007; Yu et al., 2017), the combined application of a genetically enhanced auxotroph with biochemical aconitase inhibition to redirect TCA flux for enhanced citric acid production has not been reported to our knowledge. Furthermore, unlike prior studies that primarily used UV or EMS-induced mutations (Behera, 2020), we employed HNO₂-induced mutagenesis followed by L-cysteine-based repression screening, targeting a unique metabolic bottleneck.

**References:**

- Behera, B.C. (2020). Citric acid from *Aspergillus niger*: a comprehensive overview. *Crit Rev Microbiol*, 46(6), 727–749. <https://doi.org/10.1080/1040841X.2020.1828815>
- Papagianni, M. (2007). Advances in citric acid fermentation by *Aspergillus niger*: Biochemical aspects, membrane transport and modeling. *Biotechnol Adv*, 25(3), 244–263. <https://doi.org/10.1016/j.biotechadv.2007.01.002>
- Yu, D., Shi, Y., Wang, Q., Zhang, X., Zhao, Y. (2017). Application of methanol and sweet potato vine hydrolysate as enhancers of citric acid production by *Aspergillus niger*. *Bioresour Bioproc*, 4(1):35. <https://doi.org/10.1186/s40643-017-0166-4>

**2. Experimental Design & Controls**

**There is no clear negative control for aconitase inhibition—e.g., a condition with methanol and ferrocyanide in a strain where aconitase is genetically knocked down or confirmed inactive.**

**Suggestion: Future work should include enzymatic assays or gene expression profiles for aconitase and citrate synthase activity levels to support mechanistic claims.**

**Response:** We thank the reviewer for the comment. While we did not perform aconitase activity assays or transcriptomic profiling in this study, we have now acknowledged this as a limitation in the Discussion and Conclusion sections. Future studies will include quantitative assays for aconitase and citrate synthase activities and RT-qPCR analysis to confirm the enzymatic and gene expression profiles related to the TCA cycle. This will strengthen the mechanistic understanding of aconitase co-inhibition (**Lines: 238-249; 499-510**).

**3. Strain Development**

**The mutagenesis and screening workflow is presented, but there’s no genetic validation (e.g., mutation site confirmation or genome sequencing).**

**Suggestion: At least brief mention of sequencing or molecular characterization (if not performed, note as limitation).**

**Response:** We thank the reviewer for the comment. Genetic validation via whole-genome sequencing or PCR-based mutation analysis was not conducted in this study due to resource constraints. However, the phenotypic resistance to L-cysteine HCl and enhanced citric acid productivity reliably indicate successful mutagenesis. A statement addressing this has been added to the Discussion. Future work will involve sequencing and molecular characterization to correlate phenotypic performance with genetic markers (**Lines: 238-249**).

**4. Fermentation Type Confusion**

**The manuscript interchanges “batch” and “continuous” fermentation rather loosely.**

**Clarify: Are the experiments truly continuous (with fresh medium addition/removal), or pseudo-continuous or repeated-batch? Please define this clearly.**

**Response:** We thank the reviewer for the comment. The term “continuous fermentation” in our manuscript refers to a quasi-continuous or repeated-batch fermentation, where portions of fermented broth were periodically replaced with fresh SMS medium every 24 hours after 144 h. We have now clarified this distinction in both the Materials and Methods (**Lines 152-157**) and Results sections (**Lines: 354-358**), using the term “repeated-batch” to avoid confusion.

**5. Data Interpretation**

**Kinetic data (e.g., Fig. 6 and 7) lack statistical comparison across groups and are hard to interpret without units on all figures.**

**Suggestion: Add error bars, units, and clear trend descriptions in all graphs. Also, add a table summarizing key productivity indicators across all experimental groups.**

**Response:** We thank the reviewer for the comment. As suggested, we have added full units on figure 6 and 7 and ensured that all graphs must have error bars. Additionally, a new Table 3 has been added to summarize key productivity indicators (Yp/x, Yp/s, Qp, Qx, qp, qx) across different conditions and strains. Y-Error Bars indicate standard deviation (set at 5%) among three parallel replicates (**Lines: 665-669**). Moreover, for comparison of kinetic data, Table 2 is already there.

**Minor Comments**

**Grammar, Style, and Formatting**

**The text has frequent grammatical issues, especially subject-verb agreement and article usage (e.g., “was released 2.35 g/L citric acid productivity” → should be “produced 2.35 g/L of citric acid”).**

**Phrases like “poor productivity” or “good morphology” are subjective. Use quantifiable language.
Response:** We thank the reviewer for the comment. We have thoroughly proofread and revised the manuscript to ensure:

- Correct subject-verb agreement and article usage
- Use of quantitative descriptors instead of vague terms. For example, “poor productivity” is now specified, as suggested.

**Abstract
Good summary, but it needs less technical jargon for broader accessibility. The phrase “aconitase co-inhibition strategy” should be briefly explained even in the abstract.**

**Response:** We thank the reviewer for the comment. The Abstract has been simplified to ensure broader accessibility. The term “aconitase co-inhibition strategy” is now briefly defined in the abstract (**Lines 4-21**).

**Units and Symbols**

**Be consistent with units: write “g/L” uniformly (sometimes “g/l” is used).**

**Chemical names like K₄Fe(CN)₆ should always be italicized or subscripted for readability.**

**Response:** We thank the reviewer for the comment. We have revised the manuscript thoroughly to ensure:

- Consistent use of g/L
- Subscript formatting of K₄Fe(CN)₆, NaOH, CO₂, etc., where applicable.

**Figures and Tables**

**Figure legends are often insufficient. Each should include a full description of the experimental setup (strain, medium, inhibitors, timepoints).**

**Figure 4 could be enhanced with higher resolution images or SEM visuals to better highlight morphological differences.**

**Response:** We thank the reviewer for the comment. All figure legends have been expanded to include:

- Strain names
- Inhibitor concentrations
- Timepoints

Figure 4 has been updated with improved resolution. Although higher-resolution images are limited, we have provided two versions: one zoomed-in and one zoomed-out to aid clarity and interpretation. Alternatively, if quality is still not acceptable to the editorial team, we are willing to remove this figure as per final recommendation.

**Consider a summary table comparing wild-type and NA-CYS3 under all tested conditions (batch and continuous, with and without inhibitors).**

**Response:** We thank the reviewer for the suggestion. A summary table comparing wild-type and NA-CYS3 under all conditions has been added as Table 3. We have added it here as well.

Table 3: Summary of citric acid production, yield coefficients (Yp/s), and volumetric productivity (Qp) for wild-type and NA-CYS3 strains under various tested fermentation conditions. Data represent mean values from triplicate experiments.

| **Condition** | **Strain** | **Citric Acid Yield (g/L)** | **Yp/s (g/g)** | **Qp (g/g/h)** |
| --- | --- | --- | --- | --- |
| Batch (no inhibitor) | Wild-type (IIB-4) | 2.35 | 0.12 | 0.002 |
| Batch (no inhibitor) | NA-CYS3 | 4.32 | 0.39 | 0.008 |
| Continuous (no inhibitor) | Wild-type (IIB-4) | 16.62 | 0.12 | 0.002 |
| Continuous (no inhibitor) | NA-CYS3 | 26.35 | 0.39 | 0.008 |
| Continuous (K₄Fe(CN)₆ + Methanol) | Wild-type (IIB-4) | 35.65 | 0.12 | 0.002 |
| Continuous (K₄Fe(CN)₆ + Methanol) | NA-CYS3 | 48.96 | 0.39 | 0.008 |

**References
Some references are outdated (e.g., from the 1980s or early 2000s). Recent advances in metabolic engineering and fermentation optimization could improve context.**

**Ensure DOIs are complete and verify that all references are cited correctly in-text.**

**Response:** We thank the reviewer for the comment. We have reviewed and updated the reference list with recent literature (2016–2024), particularly those focusing on metabolic engineering, aconitase inhibition, and fermentation optimization, while retaining seminal works for historical context. We have also ensured the DOI are complete and that all references are cited in-text correctly.

**References:**

- Behera, B.C. (2020). Citric acid from Aspergillus niger: A comprehensive overview. Critical Reviews in Microbiology, 46(6), 727–749. https://doi.org/10.1080/1040841X.2020.1828815
- Cairns, T.C., Zheng, X., Zheng, P., & Sun, J. (2021). Metabolic engineering and synthetic biology of Aspergillus niger: Current status and prospects. Biotechnology Advances, 52, 107794. https://doi.org/10.1016/j.biotechadv.2021.107794
- Khandagale, A.B., et al. (2021). Comparative analysis of citric acid production by Aspergillus niger using different media. Plant Cell Biotechnology and Molecular Biology, 22(3-4), 77–85.
- Mazutti, M.A., et al. (2020). Advances in the production of citric acid from biomass. Renewable and Sustainable Energy Reviews, 119, 109552. https://doi.org/10.1016/j.rser.2019.109552
- Nadeem, A., et al. (2020). Enhanced citric acid production by Aspergillus niger using low-molecular-weight alcohols. Turkish Journal of Biochemistry, 45(4), 411–419. https://doi.org/10.1515/tjb-2020-0142
- Show, P.L., et al. (2021). Valorization of waste biomass for sustainable biotechnological production of citric acid. Bioresource Technology, 319, 124229. https://doi.org/10.1016/j.biortech.2020.124229
- Xu, Y., et al. (2021). Omics-guided metabolic engineering of Aspergillus niger for enhanced citric acid production. Bioresource Technology, 320, 124353. https://doi.org/10.1016/j.biortech.2020.124353
- Yu, X., et al. (2021). Mechanistic insights into methanol-mediated citric acid overproduction in Aspergillus niger. Journal of Fungi, 7(12), 1030. https://doi.org/10.3390/jof7121030
- Yousuf, A., et al. (2022). Adaptive laboratory evolution of Aspergillus niger for improved citric acid production under oxidative stress. Fungal Biology and Biotechnology, 9, 11. https://doi.org/10.1186/s40694-022-00132-7
- Zheng, X., et al. (2022). Comprehensively dissecting the hub regulation of PkaC on high-productivity and pellet macromorphology in citric acid-producing Aspergillus niger. Microbial Biotechnology, 15(6), 1867–1882. https://doi.org/10.1111/1751-7915.14020

**Overall Recommendation: Major Revisions**

**Why? The study is promising and relevant for industrial bioprocessing, but it needs:**

- **Improved clarity in methodology and data interpretation,**
- **Stronger mechanistic support,**
- **Language polishing, and**
- **Refinement in figure/table presentation.**

**Response:** We thank the reviewer for the comment. We sincerely thank the reviewer for the constructive feedback and for recognizing the relevance and potential of our study for industrial bioprocessing applications. In response to the valuable suggestions:

- We have revised the methodology section to enhance clarity and ensure reproducibility by providing additional details (where possible) regarding experimental design, timepoints, controls, and statistical analysis.
- To address the concern regarding mechanistic support, we have expanded the discussion to better explain the biochemical rationale behind the aconitase co-inhibition strategy and its impact on metabolic flux toward citric acid accumulation.
- The manuscript has undergone extensive language polishing to improve readability, correct grammatical issues, and ensure a more consistent scientific tone.
- All figure legends have been expanded to include strain names, inhibitor concentrations, and timepoints. We have also refined table formatting and improved image presentation wherever possible. For Figure 4, although higher-resolution images were not available, we have included both zoomed-in and zoomed-out versions to improve clarity.

**Authors’ Response to Reviewer #4:**

**The manuscript titled “Enhanced Citric Acid Production via Aconitase Co-Inhibition Strategy Using L-Cysteine HCl-Resistant Aspergillus niger NA-CYS3,” coded as BITC-D-25-00797, suggests a novel biotechnological approach to increasing citric acid production. This strategy involves genetically mutating a wild-type strain of *Aspergillus niger*, along with specific enzyme inhibition through potassium ferrocyanide and methanol. While the research is highly relevant for both academic and industrial applications, some clarifications are needed to enhance comprehension and readability.**

**-Please provide recent references in the introduction section, particularly for line 45.**

**Response:** We thank the reviewer for the comment. The introduction section has been revised and the references have been updated (**Lines 39-65**). Following are the revised references:

- Behera, B. C. (2020). Citric acid from *Aspergillus niger*: a comprehensive overview. *Critical Reviews in Microbiology*, *46*(6), 727–749. <https://doi.org/10.1080/1040841X.2020.1828815>.
- Zhang, N., Jiang, JC., Yang, J., Wei, M., Zhao, J., Xu, H., Xie, JC., Tong, YJ., Yu L. (2019). Citric Acid Production from Acorn Starch by Tannin Tolerance Mutant *Aspergillus niger* AA120 *Appl. Biochem. Biotechnol*., 188(1), 1–11. <https://doi.org/10.1007/s12010-018-2902-4>.
- Zheng, X., Cairns, TC., Ni, X., Zhang, L., Zhai, H., Meyer, V., Zheng, P., Sun, J. (2022). Comprehensively dissecting the hub regulation of PkaC on high-productivity and pellet macromorphology in *Aspergillus niger*. *Microb. Biotechnol.,* 15(6), 1867–1882. <https://doi.org/10.1111/1751-7915.14020>.

These recent works reinforce the importance of *A. niger* in industrial citric acid production and highlight advances in fungal biotechnology.

**-Has the protocol for induced mutagenesis using nitrous acid been published? If so, please share the references. If not, were other concentrations of the nitrous acid solution tested? I would appreciate further details on this.**

**Response:** We thank the reviewer for the comment. Yes, the use of nitrous acid for induced mutagenesis in *A. niger* has been previously reported. We have now cited the following protocol-based reference in the Materials and Methods section:

- Justin, K., Viateur, U., & Prudentienne, M. (2010). Use of nitrous acid mutant of Aspergillus niger for citric acid production from local cane-molasses. *Afr J Microbiol Res*, 4, 1446-1452.

Additionally, we tested a range of nitrous acid concentrations (2–12 mM) to determine optimal mutation efficiency. These experimental details have been presented in Table 1 and described in the Results section.

**-Additionally, there is a lack of** **references or detailed protocols regarding the de-repression of the mutant strain. Please provide this information.**

**Response:** We thank the reviewer for the comment. We have now added an explanatory note and reference to the screening of L-cysteine HCl-resistant auxotrophs. While our specific derepression protocol was adapted from internal optimisation, a similar approach is supported by:

- Lotfy, W. A., Ghanem, K. M., & El-Helow, E. R. (2007). Citric acid production by a novel Aspergillus niger isolate: I. Mutagenesis and cost reduction studies. Bioresource Technology, 98(18), 3464-3469.
- Hanif, A., Yasmeen, A., & Rajoka, M. I. (2004). Induction, production, repression, and de-repression of exoglucanase synthesis in Aspergillus niger. Bioresource Technology, 94(3), 311-319.

The concentrations used (0.25–1 mg/L of L-cysteine HCl) were chosen to select for repressor-resistant auxotrophs and have now been justified and described with references in the revised text.

**-There is a typo in line 117.**

**Response:** We thank the reviewer for the comment. We have corrected the typographical error in line 117. Thank you for drawing our attention to it.

**-I recommend always capitalizing “Petri plate” to honor its inventor, Julius Petri.**

**Response:** We thank the reviewer for the comment. We have revised the manuscript to capitalize “Petri Plate” consistently throughout the text.

**-What volume and concentration of bromocresol green solution were added to the medium?
Response:** We thank the reviewer for the comment. We have now included the following detail in the Materials and Methods section: “Bromocresol green was added at a final concentration of 0.04% (w/v) in a volume of 1 mL per 100 mL of medium.” (**Lines 136-137)**

**-An experimental design utilizing statistical methods could help minimize the number of assays required.**

**Response:** We appreciate the reviewer’s insightful suggestion. We fully agree that employing a statistical experimental design—such as Response Surface Methodology (RSM) or Design of Experiments (DoE)—could optimize process parameters more efficiently and reduce the number of experimental trials required. While the current study was based on classical one-variable-at-a-time (OVAT) optimization, we acknowledge the advantages of multifactorial approaches in identifying interactive effects and improving experimental economy.

To reflect this, we have included a statement in the Discussion section acknowledging this limitation and have noted it as a recommendation for future work (**Lines 449-456**). We plan to integrate RSM or similar statistical tools in the next phase of this research to systematically optimize inhibitor concentrations, incubation time, and inoculum size for maximum citric acid yield.

**-Does the increase in citric acid production justify the costs associated with introducing enzyme inhibitors into the medium?**

**Response:** We thank the reviewer for the comment. This is an important industrial consideration. Our data show that the co-inhibition strategy increased citric acid yield by 1.3-fold (48.96 g/L vs. 35.65 g/L). Potassium ferrocyanide and methanol are used in very low concentrations (0.04% and 1 mL, respectively), making their cost marginal relative to the yield gains. Nevertheless, we now acknowledge in the Discussion that cost–benefit analysis at industrial scale must be performed to validate economic feasibility (**Lines 475-480**).

**-At the optimized concentration of potassium ferrocyanide (0.04% w/v), should this compound be recovered from the citric acid produced for food applications?**

**Response:** We thank the reviewer for the comment. We have explicitly stated this in Discussion section (**Lines 480-483**):

“For food-grade citric acid production, potassium ferrocyanide must be removed during downstream processing to meet safety standards. Alternative non-toxic aconitase inhibitors or food-grade extraction techniques may be explored in future work.”

**-Fig 1 needs a control medium without fungal growth, as the clear zones of glucose-hydrolysis are not visible. Please indicate them with arrows.**

**Response:** We thank the reviewer for the comment. We have now provided the control medium and tried to indicate the fungal growth in other samples with arrows in Fig. 1.

**-Fig 4 should be replaced with a better one or removed from the manuscript.
Response:** We thank the reviewer for the comment. Figure 4 has been updated with improved resolution. Although higher-resolution images are limited, we have provided two versions: one zoomed-in and one zoomed-out to aid clarity and interpretation. Alternatively, if quality is still not acceptable to the editorial team, we are willing to remove this figure as per final recommendation.

**Comments from the Executive Editor in Chief:**

Follow the instructions carefully when revising the manuscript. Following all the requirements outlined in this document will avoid a delay in the submission to decision time line.

1. Please revise the abstract. The abstract should contain a summary of the results and should exclude an introduction. The abstract must provide enough information that will allow a reader to understand the key findings of the research.

**Answer:** We thank the Editor for this valuable suggestion. The abstract has been thoroughly revised to focus exclusively on the key findings of the study. All introductory background information has been removed, and the revised abstract now clearly summarizes the major experimental outcomes, including improvements in citric acid production and the impact of enzyme inhibition.

2. The figure legends needs improvement. All legends should have enough description for a reader to understand the figure without having to refer back to the main text of the manuscript.

**Answer:** We have revised all figure legends to provide comprehensive and standalone descriptions. Each legend now includes sufficient detail to allow the figures to be understood independently of the main text. Additionally, all figures are appropriately cited and discussed within the manuscript.

3. 3Biotech does not have a copy editor for language improvements and authors are recommended to undertake steps for this task. Authors should at the minimum proof read the entire manuscript for typographical errors and fix all grammatical errors. I would strongly recommend that you request your colleagues who have a good command of the English language to assist you in the revision and also seek the services of an experienced commercial language Editor for this purpose. Normally, commercial language editing services (sometimes referred to as copy-editing), will only correct grammar and will not change the scientific expressions contained in a manuscript. However, some editorial services will also provide an additional “Substantive Editing Services”
   in which the content is reshaped to improve it’s organization and content (style, rephrasing), with the aim of improving the flow (readability) and to ensure that the language use is appropriate to the nature of the material for a specific audience (scientists, social scientists, lawyers etc). The former service costs less than the later service. Please note that the use of an editing service is at the author’s own expense and in no way implies that the article will be accepted by 3 BIOTECH. The decision of accepting a manuscript by 3 BIOTECH is based on the quality and suitability of a manuscript and is independent of whether that manuscript has been professionally edited with regard to the English language. 3 BIOTECH accepts no responsibility for the interactions between the author and the service provider or for the quality of the work performed.” For this reason, the 3 Biotech editorial board does not recommend an editorial service provider.

**Answer:** We appreciate the clarification and recommendation from the editorial board. The entire manuscript has been carefully proofread for grammatical correctness and clarity. We have also sought input from colleagues proficient in academic English to further refine the language and ensure clarity, coherence, and appropriate scientific tone.

4. Please include on a separate page a detailed and specific response / rebuttal on the revision undertaken for all the comments of the review panel and upload this page with your revised manuscript. To address a reviewers comments by stating “done” is considered to be an unacceptable response.

**Answer:** We have prepared a detailed point-by-point response to all comments from the reviewers. Each response clearly outlines the revisions made, with references to the specific changes in the manuscript and corresponding line numbers. This document has been uploaded as a separate file alongside the revised manuscript.

5. All changes made in the revised manuscript should be easily identified from the original version. I would suggest that you use a different colored font to highlight the changes that have been made but please do not submit the revised manuscript as a doc with track changes on. The handling Associate Editor and the reviewers will be able to provide a much faster response if the revised text can be easily identified by them.

**Answer:** As recommended, all revisions in the manuscript have been highlighted in red font to ensure clear visibility for the reviewers and the handling editor. The associated response file also includes line numbers to facilitate easy cross-referencing with the manuscript text.
